# On the Use of Carbolic Acid in Diptheritic Affections

**Published:** 1871-07

**Authors:** F. C. Hotz

**Affiliations:** Chicago


A MONTHLY RECORD OF

*Medicine, Surgery, and the Collateral Sciences.*

EDITED BY J. ADAMS ALLEN, M.D., LL.D.; AND WALTER HAY, M.D.

VOL. XXVIII.—JULY, 1871.—NO. 7.

### Original Communications.

ARTICLE I.—*On the Use of Carbolic Acid in Diphtheritic Affections.* By F. C. HOTZ, M.D., Chicago.

After the carbolic acid had, recently, been used so frequently and successfully in Surgery to prevent the septic infection, the natural consequence was to try its efficiency in diphtheria, whose most dangerous feature lies in the septic infection. So it was then tried here and there, but abandoned again. I wish, therefore, to draw the attention of the profession anew to this remedy, by reporting a number of cases of diphtheria which, during the last winter, I treated with the most gratifying success with carbolic acid. I used for this topical medication the acid in the following form:

| | | |
|---|---|---|
| ℞ | Acid Carbolic, cryst. - - - - - - | } āā ʒj; |
| | Alcohol, - - - - - - - - - - | } |
| | Aquæ, - - - - - - - - - - - | ℥ v; |
| | Tinct. Iodin., - - - - - - - - | ℥ ss. M. |

This makes a perfectly clear, transparent mixture, of a brown-red color, which soon, however, passes over into a pale yellow. The combination with alcohol and iodine effectually moderates the very unpleasant smell of the carbolic acid, and increases its antiseptic effect. This solution was applied to the diphtheritic exudation three or four times in twenty-four hours, by means of a camel's hair brush. In adults and older children, it was, in a diluted form (15 to 30 drops to a cup of water), used also as a frequent gargle, and for injections into the nostrils if the nose was implicated.

I was induced to try this treatment by the report of a German physician,* who saw even the most unpromising cases recover under this medication in a very short time, and as my observations could in every point confirm this experience, I thought it to be of sufficient interest and importance to communicate an account of my cases.

CASE I.

Was called, Dec. 15th of last year, to see a boy, aged 8 years, who two days previously was attacked with fever, severe pain in the throat, and swelling of the cervical glands. Found the tonsils very large, and covered with patches of white exudation. A saline purgative and a gargle of alum was given. But on the following day, Dec. 16th, the patient was in a half soporous state, pulse 140, skin hot and dry; breath fœtid, and a thin offensive fluid was discharged from the nostrils. The tonsils were so large that they nearly closed the isthmus of the fauces. The soft palate and the tonsils were covered with a thick white exudation firmly adherent to the tissue. Mucous membrane of the pharynx deep red, and covered with viscid mucus.

The mixture was applied four times, and diluted with water (20 drops to a teacup) it was frequently used as a gargle and injection into the nostrils. An infusion of digitalis was also given.

Dec. 17th.—The patient conscious; a. m. pulse 115; p. m. 100;

* Dr. Rothe, in *Berliner Klinische Wochenschrift*, June 13, 1870. He reports fifteen cases: in two (children aged 3 and 7 years) the diphtheria was complicated with scarlatina; in one (child of 3 years) with the measles; in five children, from 1½ to 7 years old, the disease had extended to the larynx; in six cases the doctor arrested the disease right in the beginning. All cases did well. Digitalis was given in the acme of the fever; liquor ferri muriatis in the reconvalescence.

the discharge from the nose thicker and inoffensive; the exudation thinner and not extended any further. Digitalis discontinued.

Dec. 18th.—Pulse 96; very little discharge from the nose; exudation only on the uvula; cervical glands diminished in size. Mixture twice more applied to the uvula, and then discontinued. Gargle continued for two days longer, when the patient was discharged cured.

CASE II.

Saw, Dec. 24th, the servant girl in the same family, who was taken sick the previous night with chill and fever, headache and pain in the throat. Pulse 96; tonsils very tender to the touch, enlarged, and covered with patches of white exudation; mucous membrane of soft palate and pharynx deep red, and covered with thick viscid mucus.

The mixture was applied to the tonsils twice daily, and the gargle used every hour. The patient recovered in two days.

CASE III.

Adult in the same house was, Dec. 25th, affected in the same manner as Case II, and under the same treatment he recovered in two days.

CASE IV.

A boy, 5 years old, was attacked with scarlatina, and nothing abnormal occurred during the eruptive stage (Dec. 18th to 22nd), but on the 23rd of Dec. the fever increased in intensity; the cervical glands were swollen; a thin mucus streaked with blood was discharged from the nose, and the boy complained of pain in the throat.

The diluted mixture only was used as injections into the nostrils every two hours.

Dec. 24th—a. m. pulse 125, p. m. 140, the patient unconscious; swallowing impeded, lips and tongue dry; tonsils greatly enlarged and thickly covered with diphtheritic exudation. The same exudation extended over the soft palate and pharynx. Mixture applied three times; injections continued.

Dec. 25th—Pulse 120; the boy decidedly better; conscious, can swallow, though with difficulty; tongue moist; pharynx already freed from exudation; discharge from the nose diminished in quantity and of thicker consistence. Enlargement of the cervical glands diminished.

Dec. 27th—Pulse 96; the patient has good appetite, swallows without difficulty or pain; discharge from the nose has ceased; mucous lining of pharynx and tonsils natural. The patient discharged cured.

CASE V.

An adult, aged 32 years, was, on 3rd of April, attacked with severe fever and stinging pain in the throat; tonsils very tender, enlarged and covered with patches of white, firm exudation (as in Case II,) which also extended over the whole uvula. With two applications of the mixture, and by frequent gargle with the diluted solution, the patient recovered in three days.

CASE VI.

Was called, April 9th, to see a child, 15 months old, who had contracted the measles from a sister. Nothing unusual occurred until the 6th of April (which was the second day of the eruption), when the right eyelids rapidly became swollen, and on the following day the left lids also commenced to be œdematous. On these three days previous to my first visit, the child was very feverish, slept much, and refused to swallow any food. Found, April 9th, the child unconscious, and with high fever. The respiration was difficult, and the cervical glands were much swollen. There was a thin offensive discharge from the nose, whose mucous lining appeared infiltrated with a firm fibrinous exudation. The upper lip swollen and hard, its skin red and glistening, and to a great extent destroyed by diphtheritic ulceration. The tonsils enlarged and covered with thick exudations (partially brown—red from blood), as were also the soft palate and the pharynx. The right upper eyelid was swollen to the size of an egg, so hard and stiff that it was impossible to raise it sufficiently to see the condition of the cornea. As far as the conjunctiva could be seen it was thick and pale from a gelatino-fibrinous exudation. The edge of the right upper lid was partially destroyed by ulceration. The left eyelids were less swollen and hard than the right, their conjunctiva deep red, but smooth, and covered with patches of exudation; the middle of the free edge of the left upper lid was also destroyed by diphtheritic ulceration.

Mixture applied to the conjunctiva as well as to the fauces three times daily, and, diluted (30 drops to a tumbler of water), it was used as a mouth wash and injection into the nostril every hour.

April 10th—Left lids very little swollen; conjunctiva free from exudation. Swelling and stiffness of the right lids also diminished; the diphtheritic exudation thinner, so that the conjunctiva has a pale rosy tint; respiration easier. Treatment continued.

April 12th—No discharge from the nose, its mucous membrane natural; the ulcer of the upper lip healing; the exudation in the fauces very thin; left eye natural; conjunctiva of the right lids (which were soft and of natural size) free from exudation, but red and succulent. The orbicular conjunctiva still showed a thin exudation, and the cornea was opaque, necrotic. Mixture twice.

April 14th—Mouth and fauces clear; also right eye freed from the rest of diphtheritic exudation; but the cornea was totally destroyed. Treatment discontinued.

I saw the child on the 19th again; it had recovered pretty fairly, and all traces of that severe disease had disappeared except the destruction of the right eye, some catarrhal ophthalmia, and great weakness. During the whole sickness good nourishment was all the child took internally.

The following two cases have been under the care of Dr. Mannheimer, to whom I am indebted for their history.

### CASE VII.

Was, October 28th, called to see a boy, aged 4 years, who, two days previously, was taken sick with high fever, and severe pain in the throat. Found the cervical glands swollen, and a thin but not offensive discharge from the nose; and diphtheritic exudation covering the lips, tonsils, soft palate and pharynx. The mixture was applied three times daily, and the gargle used frequently. After two days the patient was so much better, that one topical application only was required daily; and after four days more the boy was discharged cured.

### CASE VIII.

Saw, in November, a child 2 years old, who was suffering from the same diphtheritic affection of the tonsils, soft palate and pharynx, as Case VII; but complicated by an extension of the exudation to the larynx as a complete aphony, and very difficult respiration indicated. The same treatment as in the previous case was adopted, with the addition that, with a suitable brush, the mixture was applied to the larynx also until, after two days, the voice returned and the respiration was easy. Discharged as cured after ten days.

These cases are not very numerous, but they suffice, in my opinion, to show that the local treatment which was applied had an evident influence on the diphtheritic inflammation. In all cases, the mixture at once arrested the extension of the exudation, and where it was used in the stage of the development, the disease was actually cut short. Although the Cases II, III and V, in themselves alone, are not conclusive evidence for this assertion, because one may doubt their diphtheritic nature, yet they are of great value in connection with Case I. Occurring at about the same time, some in the same house, and all beginning with the same local and general symptoms, they showed this remarkable difference, that in Case I the disease, when let alone, rapidly developed all signs of a severe diphtheritic inflammation, while it passed, as a perfectly harmless affection, in Cases II, III and V, in which the mixture was used at once. And then, I refer to Case VI as the most striking instance; there the diphtheria was fully developed, not only in the throat but also in the right eye, and, the contagious matter being transmitted to the other eye, this was threatened with incipient diphtheritic ophthalmia. But within two days this eye was out of danger, as the inflammation was arrested in its earliest stage, owing to the timely use and the effectual action of the above mixture.

Nor is the efficiency of this remedy less strikingly demonstrated by those severe Cases (Nos. IV, VI and VIII) where the diphtheria was far advanced before the above topical treatment was resorted to. In those cases even, I succeeded always in checking the local inflammation instantly, and soon after the constitutional symptoms subsided also. We further noticed that under the use of the mixture the exuded matter rapidly diminished in quantity and disappeared, but putrefaction of the exudation, and gangrenous destruction of the mucous membrane never occurred after the remedy had been applied.

I lay great stress upon these observations, as they clearly show that the constitutional disturbance is closely connected with, and entirely dependent on, the local inflammatory process. Just as the general symptoms accompanying an acute inflammation of a joint will assume a very dangerous character when the exudation in the joint becomes purulent or putrid, in the same manner the diphtheria will seriously affect the constitution when the exudation undergoes putrid decomposition. And the beneficial effect of the above treatment consists, in my opinion, in the prevention of the occurrence of such putrefaction, and in thereby protecting the system from putrid infection, and the mucous membranes from extensive ulceration.

I am well aware that such exclusive topical treatment is strongly objected to by those who consider diphtheria to be a constitutional disease. Though a wide-spread opinion, its correctness has not yet been fully evinced by pathological facts; while, on the other hand, there are many observations which do not speak in its favor. I will mention but a few facts. When one eye is affected with diphtheritic conjunctivitis, we can protect the other eye against contagion, by a bandage applied with the utmost care and accuracy, while internal remedies are of no avail. Nor can such remedies prevent the exudations spreading to the larynx, while we can keep them confined to the pharynx by local applications. And in all records on diphtheria experience has taught that local medication yielded altogether better results than the mere internal administration of medicines. By these and other facts we are well justified in searching for a reliable local remedy in the treatment of diphtheria, though such treatment may not be in harmony with our theory. For I believe the aim of our professional labor to be to investigate into the nature of diseases, but not to find out proofs for an assumed theory.

